# Drug Retention Rates and the Safety of Janus Kinase Inhibitors in Elderly Patients with Rheumatoid Arthritis

**DOI:** 10.3390/jcm12144585

**Published:** 2023-07-10

**Authors:** Jumpei Temmoku, Masayuki Miyata, Eiji Suzuki, Yuya Sumichika, Kenji Saito, Shuhei Yoshida, Haruki Matsumoto, Yuya Fujita, Naoki Matsuoka, Tomoyuki Asano, Shuzo Sato, Kiyoshi Migita

**Affiliations:** 1 Department of Rheumatology, Fukushima Medical University School of Medicine, 1 Hikarigaoka, Fukushima 960-1295, Fukushima, Japan; temmoku@fmu.ac.jp (J.T.); ysumiti@fmu.ac.jp (Y.S.); s3xbck2p@fmu.ac.jp (K.S.); shuhei-y@fmu.ac.jp (S.Y.); haruki91@fmu.ac.jp (H.M.); fujita31@fmu.ac.jp (Y.F.); naoki-11@fmu.ac.jp (N.M.); asanovic@fmu.ac.jp (T.A.); shuzo@fmu.ac.jp (S.S.); 2 Department of Rheumatology, Jananese Red Cross Fukushima Hospital, Yashima 7-7, Fukushima 963-8558, Fukushima, Japan; fukuintyoumm@fukushima-med-jrc.jp; 3 Department of Rheumatology, Ohta-Nishinouchi Hospital, 2-5-20 Nishinouchi, Koriyama 963-8558, Fukushima, Japan; azsuzuki@ohta-hp.or.jp

**Keywords:** janus kinase inhibitor, elderly patient, drug retention rate, safety, adverse events

## Abstract

Background: We examined the real-world drug retention rate and safety data of Janus kinase inhibitors (JAKis) in elderly patients with rheumatoid arthritis (RA). Methods: This study enrolled 133 RA patients (≥65 years) with sufficient clinical data who were initiated with JAKis during the study period. These patients were divided into two groups: the very elderly group (≥ 75 years) and the elderly group (65 ≤ years < 75). The drug retention rates of JAKis were compared using Kaplan–Meier curves. Results: The discontinuation rates of JAKis were as follows: lack of effectiveness 27 (20.3%), adverse events (AEs) 29 (21.8%), and remission 2 (1.5%). There was no significant difference in the overall drug retention rate between the very elderly group (≥75 years) and the elderly group. Furthermore, the overall drug retention rates of JAKis were not affected by gender, methotrexate use, and anti-citrullinated protein/peptide antibody (ACPA) status. The discontinuation rates of JAKis due to AEs were comparable both in the very elderly group (≥75 years) and the elderly group (65 ≤ years < 75). Whereas chronic lung disease and hypoalbuminemia were independently associated with discontinuation rates due to AEs, the overall drug retention rates were significantly lower in patients treated with the approved dose of JAKis than in those treated with a reduced or tapered dose. Conclusions: Our results suggest that the overall drug retention rate of JAKis in very elderly patients (≥75 years) was comparable with that in elderly patients (65 ≤ years < 75). The discontinuation rates of JAKis due to AEs were also comparable both in very elderly group patients and elderly patients.

## 1. Introduction

The Janus kinase (JAK)-signal transducer and activator of the transcription (STAT) pathway was involved in the signal transduction of several cytokine receptors [1]. The inhibition of JAKs appears to be a promising strategy for treating autoimmune diseases [2]. To date, JAK inhibitors (JAKis) are effective in treating autoimmune diseases, including rheumatoid arthritis (RA) [3]. Clinical trial data have shown comparable efficacy and safety between JAKis and biological disease-modifying anti-rheumatic drugs (bDMARDs) for treating RA [4]. Elderly patients with RA have different characteristics compared to non-elderly patients [5]. The JAKis tend to be introduced in elderly patients who are intolerant to methotrexate (MTX) due to comorbidities [6]. However, few reports have examined the real-world treatment outcomes or safety of JAKis in elderly patients with RA. It is difficult to apply the therapeutic evidence obtained from non-elderly patients to elderly patients since the increase in comorbidities and treatment-related risks may not be comparable between non-elderly and elderly patients with RA [7].

In elderly patients with RA who received JAKis, the most frequent serious adverse event was an infection, including herpes zoster, with higher risk ratios than those of young patients [8]. Recently, the Food and Drug Administration and European Medicines Agency issued warnings regarding the risk of major adverse cardiovascular events (MACE) [9]. These warnings highlighted how individuals aged more than 50 years with concomitant cardiovascular risk factors are a high-risk population for adverse events (AEs), including thrombosis [9]. The latest EULAR recommendations state that risk factors, including age over 65 years, hypertension, and a history of malignancy, should be evaluated when using JAKis [10]. It is interesting to investigate the drug retention rates of JAKis in elderly patients with RA because retention rates can be influenced by both the effectiveness and safety of molecularly targeted therapy. This study investigated drug retention and the reasons for discontinuation of JAKis among elderly patients with RA in a real-world setting using an observational multicenter retrospective cohort study. We also investigated the factors associated with drug discontinuation due to a lack of effectiveness or AEs in elderly patients with RA.

## 2. Materials and Methods

### 2.1. Patients and Study Design

We conducted a multicenter retrospective cohort study to evaluate the retention rate of JAKis and factors affecting the discontinuation of JAKis in elderly patients with RA. Our cohort consisted of patients who were treated at the Department of Rheumatology at Fukushima Medical University Hospital, Japanese Red Cross Fukushima Hospital, and Ohta Nishinouchi Hospital. Between June 2015 and July 2021, 184 patients with RA were initiated with JAKis. Among them, 133 elderly (≥65 years) patients were initiated with JAKis in our institution, and those with sufficient clinical data were enrolled in this study. All patients were diagnosed with RA according to the 2010 ACR/European League Against Rheumatism classification criteria for RA [11]. The study was approved by the Institutional Review Boards of Fukushima Medical University (No. 2019-097), Japanese Red Cross Fukushima Hospital (No. 55), and Ohta Nishinouchi Hospital (No. 2022–8). 

### 2.2. Clinical Evaluations

At the start of JAKis treatment, baseline data were collected from medical records, including demographics, clinical data (disease duration, presence of the anti-citrullinated protein/peptide antibody [ACPA] antibody, and rheumatoid factor [RF]), the evaluation of disease activity (swollen joint count, tender joint count, patient global assessment, and physician global assessment), C-reactive proteins [CRP], and information on treatments (current glucocorticoid ((GC)) and MTX doses, and previous use of bDMARDs). This treatment was at the discretion of the attending physician based on the clinical conditions and the patient’s intentions. Chronic lung disease included interstitial lung disease, bronchiolitis, bronchiectasis, and pulmonary emphysema, which were diagnosed according to abnormal findings on chest computed tomography. Hypoalbuminemia was defined as a serum albumin (Alb) level of less than 3 g/dL as an indicator of malnutrition.

### 2.3. Follow-Up

Drug retention was retrospectively evaluated as the duration until definitive treatment was interrupted. If the treatment was discontinued, the reasons for discontinuation were recorded. The decisions to discontinue JAKis were carefully determined by physicians who were board certified in the Japan College of Rheumatology based on the evaluation of clinical findings, laboratory data, and radiological examinations. The reasons for discontinuation were classified into three major categories [12]: (1) a lack of effectiveness (including primary and secondary); (2) AEs (infection, malignancies, hematological disorder, exacerbation of interstitial pneumonia, renal impairment); and (3) disease remission. Physicians were allowed to cite only one reason for discontinuation.

### 2.4. Statistical Analysis

Continuous variables are shown as the mean ± standard deviation, and categorical variables are expressed as frequencies (percentages). Drug retention was analyzed using Kaplan–Meier plots and assessed using the log-rank test. Predictive factors affecting the discontinuation of JAKis were evaluated using the univariate and multivariate analysis by the Cox proportional hazard model with SPSS Statistics software (version 25.0; IBM Corp., Armonk, NY, USA). The p-values on entering the variables for the multivariate Cox proportional hazard model were <0.1. Some minor missing baseline data, such as disease activities, were extracted by the last observation carried forward and were excluded from the adjustment confounders. Two-tailed *p* values < 0.05 were considered indicative of statistical significance.

## 3. Results

### 3.1. Baseline Characteristics of Elderly Patients with RA

**Figure 1:**
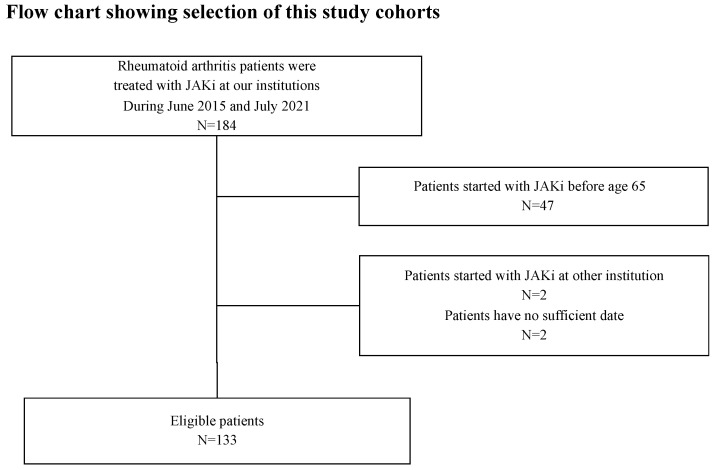
Flow chart showing the patient selection of study cohorts. Among 184 patients with RA who were initiated with JAKis at our institutions between June 2015 and July 2021, 133 elderly patients with RA and sufficient clinical data were enrolled in this study. RA, rheumatoid arthritis; JAKis, janus kinase inhibitor.

Among 184 patients with RA who were initiated with JAKis at our institutions between June 2015 and July 2021, 133 elderly patients with RA were enrolled in this study (Figure 1). The demographic and disease-related characteristics of the patients are shown in Table 1. A total of 133 patients were treated with JAKis (tofacitinib, 30; baricitinib, 74; upadacitinib, 24; and peficitinib, 4; filgotinib, 1). All patients had no prior history of JAKis use. The majority (57.1%) were women, with a mean age of 77.0 ± 7.7 years at cohort entry. The mean disease duration was 8.4 ± 10.9 years, RF positivity was 65.4%, and ACPA positivity was 63.8%. The mean of the DAS28-CRP, DAS28-ESR, and CDAI were 3.76 ± 1.12, 4.67 ± 1.31, and 20.7 ± 13.3, respectively. The mean dose and ratio of concomitant medications were GC 3.4 ± 22.3 mg/day (21.8%), and MTX was 6.5 ± 2.2 mg/week (49.0%). Forty-nine (36.8%) patients had a history of bDMARD use. As comorbid conditions, diabetes, chronic lung disease, and hypoalbuminemia (Alb < 3.0 g/dL) at the initiation of JAKis were 25 (18.8%), 30 (22.6%), and 25/124 (20.2%) patients, respectively. The mean follow-up period after initiation of JAKis was 27.4 ± 15.8 months.

### 3.2. Reasons and Rates of Drug Discontinuation

**Figure 2:**
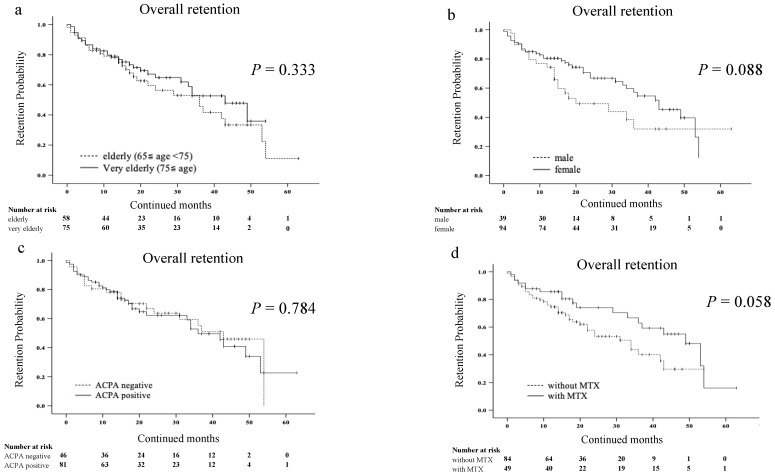
Kaplan–Meier curve related to the overall drug retention rate of JAKis in elderly patients with RA. (**a**) Between the elderly (65 ≤ years < 75) group and very elderly (≥75 years) group. (**b**) Between the male group and female group. (**c**) Between the ACPA-negative group and ACPA-positive group. (**d**) Between the group without and with MTX. The overall drug retention rates of JAKis were not affected by age, gender, ACPA seropositivity and the concomitant use of MTX. JAKis, janus kinase inhibitor; RA, rheumatoid arthritis; ACPA, anti-citrullinated protein/peptide antibody; MTX, methotrexate.

The discontinuation rates for the corresponding reasons were as follows: a lack of effectiveness 27 (20.3%); AEs 29 (21.8%); and remission 2 (1.5%). Details of the adverse events are shown in Table 2. We compared the overall drug retention rates between very elderly patients (≥75 years) and elderly patients (65 ≤ years < 75) using the Kaplan–Meier curve (Figure 2a). There was no significant difference in the overall drug retention rate of JAKis between the two groups. Similarly, the overall drug retention rates were not affected by gender, ACPA seropositivity, and the concomitant use of MTX (Figure 2b–d). 

### 3.3. Drug Discontinuation Rate Due to AEs

**Figure 3:**
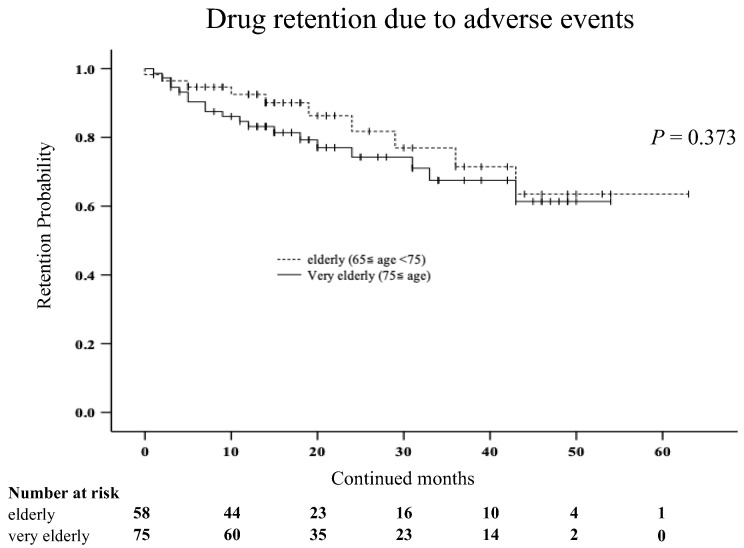
Kaplan–Meier curve related to the drug retention rate due to AEs between the very elderly group (≥75 years) and elderly group (65 ≤ years < 75) in elderly patients with RA. There was no significant difference in the discontinuation rates due to AEs between the two groups.

We compared the discontinuation rates due to AEs between the very elderly group (≥75 years) and the elderly group (65 ≤ years < 75) using Kaplan–Meier curves (Figure 3). There was no significant difference in the discontinuation rates due to AEs between the two groups.

**Figure 4:**
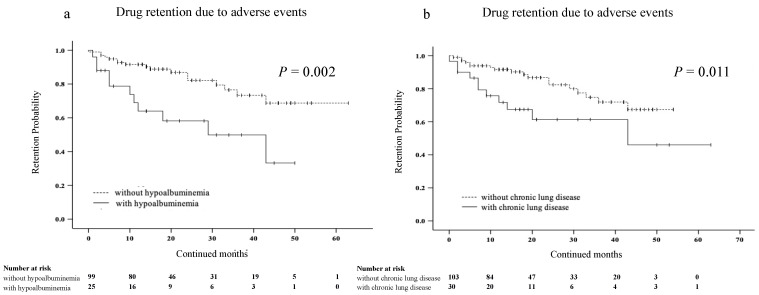
Kaplan–Meier curve related to the overall drug retention rate due to AEs in elderly patients with RA. (**a**) Between the group without and with hypoalbuminemia. (**b**) Between the group without and with chronic lung disease. The discontinuation rates due to AEs were significantly higher in the group with hypoalbuminemia than in the group without hypoalbuminemia. Similarly, the discontinuation rates due to AEs were significantly higher in patients with chronic lung disease than in those without chronic lung disease. JAKis, janus kinase inhibitor; RA, rheumatoid arthritis; AEs, adverse events.

Furthermore, we investigated several factors affecting discontinuation due to AEs using Cox proportional hazards modeling (Table 3). Univariate analysis showed that the age, the absence of MTX, the presence of chronic lung disease, and hypoalbuminemia at the time of JAKis initiation were associated with discontinuation rates due to AEs in elderly patients with RA. In multivariate analysis, the presence of chronic lung disease and hypoalbuminemia were significantly associated with discontinuation rates due to AEs in elderly patients with RA. We also compared the discontinuation rates due to AEs in the presence or absence of hypoalbuminemia or chronic lung disease using Kaplan–Meier curves. The discontinuation rates due to AEs were significantly higher in patients with hypoalbuminemia than in those without hypoalbuminemia (Figure 4a). Similarly, the discontinuation rates due to AEs were significantly higher in patients with chronic lung disease than in those without chronic lung disease (Figure 4b). 

**Figure 5:**
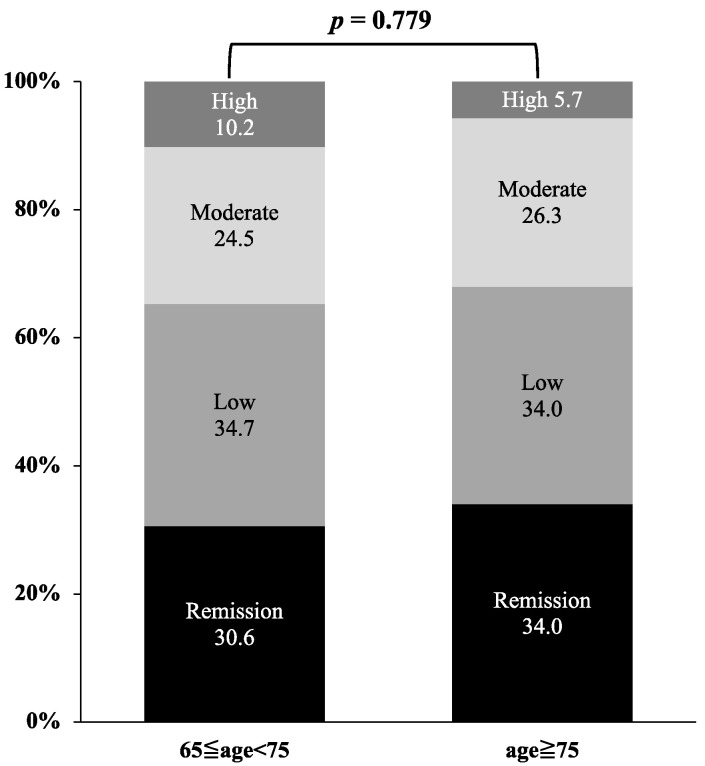
Comparison of disease activity based on DAS28-CRP between the very elderly group (≥75 years) and elderly group (65 ≤ years < 75). There were no significant differences in the percentages of low disease activity and remission at 6 months after the initiation of JAKis between the two groups.

We compared RA disease activity based on DAS28-CRP between very elderly patients (≥75 years) and elderly patients (65 ≤ years < 75) (Figure 5). There were no significant differences in the percentages of low disease activity and remission at 6 months after the initiation of JAKis between the two groups (*p* = 0.779).

### 3.4. Drug Retention Rates and Types or Dosage of JAKis

**Figure 6:**
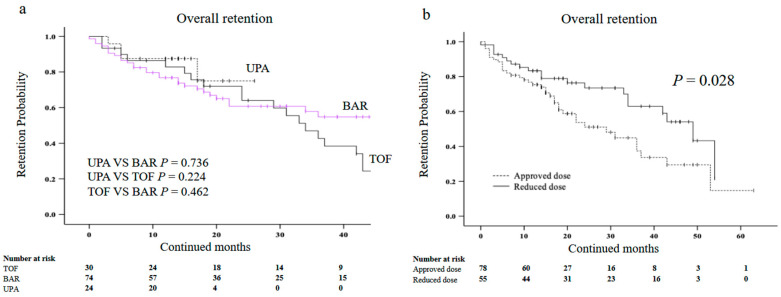
Kaplan–Meier curve related to the overall drug retention rate of JAKis in elderly patients with RA. (**a**) Between UPA and BAR, or TOF. (**b**) Between the approved dose group and reduced dose group. The retention rates appeared to be higher in the UPA group compared with the BAR group and the TOF group but there was no significant difference. The overall drug retention rates were significantly lower in the group treated with the approved dose than in the group treated with a reduced dose. JAKis, janus kinase inhibitor; RA, rheumatoid arthritis; UPA, upadacitinib; BAR, baricitinib; TOF, tofacitinib.

We also compared the overall drug retention rates among each type of JAKis (tofacitinib, baricitinib, and upadacitinib) using Kaplan–Meier curves. Although the overall retention rates of upadacitinib appeared to be higher among the three groups, there were no significant differences (Figure 6a). Finally, we investigated whether the dosage of JAKis affected the overall drug retention rates. More than half of the patients (58.6%) started with JAKis and used the approved dose continuously, while the remaining patients (41.4%)started with JAKis with a reduced dose or tapered dose during therapy on the concerns for AEs. The reduced dose was defined as any reduction in the dose, such as half doses or dosing every other day. The clinical background of patients in the reduced-dose group and the approved dose group are shown in Table 4 There were no significant differences in the clinical background between the two groups, except for age. The overall drug retention rates were significantly lower in patients treated with the approved dose of JAKis than in those treated with a reduced or tapered dose (Figure 6b). 

## 4. Discussion

Elderly patients with RA frequently experience high levels of disease activity and functional disability, which could be linked to age-related comorbidities [13,14]. The efficacy of JAKis appeared to be similar to that of bDMARDs [4], but its safety may be attenuated by aging [15]. bDMARDs and JAKis appear effective in elderly patients that are similar to younger patients [15,16]. Although the safety of JAKis can be modulated by age-related factors, which could be a concern when treating elderly patients, safety data for elderly patients with RA are limited. Therefore, drug retention rates of JAKis in elderly patients are essential.

In this study, we report the real-world drug retention and safety of JAKis in elderly patients with RA. In this observational study of elderly (≥65 years) patients with RA, we investigated the drug retention rates and safety of JAKis in elderly patients with RA. In this study, we compared the drug retention rates in very elderly patients (≥75 years) to those in elderly patients (65 ≤ years < 75); however, the drug retention rates were similar in these patients regardless of age. This is the first report demonstrating that drug retention rates were equivalent to JAKis in very elderly patients (≥75 years) compared to elderly patients (65 ≤ years < 75) in a real-world setting. Furthermore, the rates of discontinuation due to a lack of effectiveness were lower in very elderly patients (≥75 years) than in elderly patients (65 ≤ years < 75).

The effects of the ACPA serologic status on the treatment outcomes of particular bDMARDs have been demonstrated in patients with RA [17]. However, there was no significant difference in the drug retention rates of JAKis between ACPA-seropositive and seronegative elderly patients with RA. The indication for MTX should be determined carefully, considering MTX contraindications, such as renal function or pulmonary complications, in elderly patients with RA [18]. Although this could be attributed to unmeasured confounding factors, our data demonstrated that drug retention rates were not affected by the concomitant use of MTX. In accordance with our results, several studies have demonstrated the efficacy and safety of JAKis as monotherapy [19,20,21].

In our study, only half of the patients (aged ≥ 65 years) received the approved dose of JAKis, and the remaining patients received reduced or tapered doses of JAKis. However, drug retention rates were relatively higher in patients who received reduced or tapered doses of JAKis than in those receiving an approved dosage. A subgroup analysis within the RA-Beyond study examined dose reduction from 4 mg to 2 mg baricitinib in patients who achieved clinical disease control, and most patients could retain disease control after dose tapering [22]. In the JCR guidelines, dose reduction with a JAKis was conditionally recommended for patients with RA who maintained the remission or LDA after the initial dose [23]. The pharmacokinetic adjustment of the dose of JAKis should be considered in elderly patients with RA and renal or hepatic impairment. Further studies are required to determine the optimal dose for elderly patients with various comorbidities.

Recently, JAKis was reported to be associated with an increased risk of thromboembolism [24]. In our study, the incidence of cardiac vascular disorder-related potential AEs was relatively low (0.75%) in elderly patients. A recent meta-analysis also reported that JAKis did not significantly change the cardiovascular risk [25,26]. However, continuous long-term monitoring was necessary to elucidate the correlation between JAKis and cardiovascular outcomes in elderly patients because of the limited follow-up period in our study.

Our study has several limitations. First, this was a retrospective observational study with inherent biases that may have affected drug retention or discontinuation rates. Second, the choice of treatment and decision to discontinue treatment was made at the discretion of each rheumatologist, with no standardized protocol. Third, the sample size was relatively small. Fourth, the initial dose of each JAKis was determined according to the manufacturer’s recommendations, and changes in the initial dose of each JAKis were made according to the comorbidities of each patient, including CKD or the therapeutic decisions of each participating rheumatologist. Finally, patients with prior JAKis-used patients were not included in this study. 

## 5. Conclusions

The results of our study demonstrated that drug retention rates were not affected by sex, the concomitant use of MTX, or seropositivity for ACPA in real-world elderly patients with RA (≥65 years). Our results suggest that the overall drug retention rate of JAKis in very elderly patients (≥75 years) was comparable with those in elderly patients (65 ≤ years < 75). The discontinuation rates of JAKis due to AEs were also comparable both in the very elderly group of patients and elderly patients.

## Figures and Tables

**Table 1:** Baseline clinical characteristics in elderly RA patients treated with JAKis.

Characteristic	*n* = 133
Age (years)	77.0 ± 7.7
Female, n (%)	76 (57.1)
JAKis use, n	TOF 30, BAR 74, UPA 24, PEF 4, FIL 1
Disease duration (years)	8.4 ± 10.9
RF-positive, n (%)	83/127 (65.4)
ACPA-positive, n (%)	81/127 (63.8)
CRP (mg/dL)	3.12 ± 22.2
MMP-3 (ng/mL)	338.3 ± 493.7
DAS28-CRP	3.76 ± 1.12
DAS28-ESR	4.67 ± 1.31
CDAI	20.7 ± 13.3
eGFR (mL/min)	71.9 ± 22.1
CKD (eGFR < 60), n (%)	35 (27.7)
Alb (g/dL)	3.59 ± 0.60
Hypoalbuminemia (Alb < 3.0), n (%)	25/124 (20.2)
Diabetes, n (%)	25 (18.8)
Chronic lung disease, n (%)	30 (22.6)
Interstitial lung disease, n (%)	18 (13.5)
Bronchiolitis, n (%)	4 (3.0)
Bronchiectasis, n (%)	6 (4.5)
Pulmonary emphysema, n (%)	2 (1.5)
MTX use, n (%)	49 (36.8)
MTX dose (mg/week)	6.5 ± 2.2
GC use, n (%)	29 (21.8)
GC dose (mg/day)	3.4 ± 22.3
Prior bDMARDs use, n (%)	49 (36.8)
TNFi use, n (%)	34 (25.7)
Anti-IL-6R, n (%)	27 (20.3)
CTLA4-Ig, n (%)	11 (8.3)
Follow up periods (month)	27.4 ± 15.8

RA: rheumatoid arthritis; JAKis: Janus kinase inhibiter; IQR: interquartile range; TOF: tofacitinib; BAR: baricitinib; UPA: upadacitinib; PEF: peficitinib; FIL: filgotinib; RF: rheumatoid factor; ACPA: anti-citrullinated peptide antibody; CRP: c-reactive protein; DAS28: disease activity score 28; CDAI: clinical disease activity index; eGFR: estimated glomerular filtration rate; CKD: chronic kidney disease; csDMARDs: conventional synthetic disease modifying anti-rheumatic drugs; MTX: methotrexate; GC: glucocorticoid; bDMARDs: biological disease modifying anti-rheumatic drugs; TNF: tumor necrosis factor inhibiter; IL-6R: interleukin-6 receptor; CTLA4-Ig: cytotoxic T-lymphocyte associated protein 4 immunoglobulin.

**Table 2:** AEs lead to JAKis discontinuation in elderly RA patients.

Total	*n* = 29
Infection	12 (Pneumonia 8, Pyothorax 1, Upper respiratory infection 1, Herpes Zoster 1, Cholecystitis 1)
Malignancy	9 (Lymphoma 3, Lung 2, Colon 3, Melanoma 1)
Hematological disorder	4 (Anemia 2, Thrombocytopenia 1, Lymphopenia 1)
Exacerbation of interstitial pneumonia	2
Cardiovascular disease	1 (Cerebral hemorrhage)
Renal impairment	1

AEs: adverse events; JAKis: Janus kinase inhibiter; RA: rheumatoid arthritis.

**Table 3:** Cox proportional hazard analysis of the risk factors for treatment discontinuation due to AEs.

	AEs *n* = 29	Others *n* = 104	Univariable Analysis HR (95% CI) *p*-Value	Multivariate Analysis HR (95% CI) *p*-Value
Age (years)	80.0 ± 9.1	76.7 ± 7.1	1.056 (1.005–1.109) 0.031 *	1.035 (0.976–1.098) 0.249
Very elderly (age ≥ 75) (%)	19 (65.5)	56 (53.8)	1.412 (0.656–3.038) 0.377	
Female, n (%)	18 (62.1)	76 (73.1)	0.590 (0.277–1.253) 0.169	
RF-positive, n (%)	15/26 (57.7)	68/101 (67.3)	0.893 (0.408–1.957) 0.778	
ACPA-positive, n (%)	14/26 (53.8)	67/101 (66.3)	0.743 (0.343–1.611) 0.452	
CKD (eGFR < 60) (%)	7 (24.1)	28 (26.9)	1.074 (0.455–2.537) 0.870	
CRP (mg/dL)	3.1 ± 3.5	3.3 ± 4.2	1.006 (0.908–1.114) 0.915	
Hypoalbuminemia (%)	11 (37.9)	14 (13.5)	3.158 (1.478–6.750) 0.003 *	2.709 (1.201–6.112) 0.016 *
Diabetes, n (%)	6 (20.7)	23 (22.1)	0.951 (0.387–2.339) 0.913	
Chronic lung disease (%)	11 (37.9)	19 (18.3)	2.544 (1.198–5.401) 0.015 *	2.517 (1.150–5.507) 0.021 *
Reduced dose (%)	12 (41.4)	43 (41.3)	0.759 (0.359–1.602) 0.469	
MTX use, n (%)	7 (24.1)	42 (40.4)	0.407 (0.171–0.967) 0.042 *	0.536 (0.188–1.527) 0.243
GC (≥5 mg/day) use (%)	5 (17.2)	13 (12.5)	2.073 (0.784–5.481) 0.142	
Prior bDMARDs use, n (%)	12 (41.4)	37 (35.6)	1.162 (0.554–2.435) 0.691	

AEs: adverse events; HR: Hazard ratios; AEs: adverse events; RF: rheumatoid factor; ACPA: anti-citrullinated peptide antibody; CKD: chronic kidney disease; CRP: c-reactive protein; bDMARDs: biological disease modifying anti-rheumatic drugs; MTX: methotrexate; GC: * *p* < 0.05.

**Table 4:** Comparison of characteristics between the approved dose group and reduced dose group in elderly RA patients.

	Approved Dose (*n* = 78)	Reduced Dose (*n* = 55)	*p*-Value
Age (years)	76.1 ± 7.9	79.3 ± 6.9	0.014 *
Female, n (%)	53 (67.9)	41 (74.5)	0.411
Disease duration (years)	7.8 ± 10.5	9.3 ± 11.4	0.337
RF-positive, n (%)	48/74 (64.9)	35/53 (66.0)	0.891
ACPA-positive, n (%)	46/74 (62.2)	35/53 (66.0)	0.654
CRP (mg/dL)	3.2 ± 3.8	3.0 ± 3.4	0.712
DAS28-CRP	3.8 ± 1.0	3.7 ± 1.3	0.414
CDAI	22.2 ± 13.3	18.3 ± 13.3	0.155
eGFR (mL/min)	68.9 ± 21.9	76.2 ± 21.9	0.061
Hypoalbuminemia (Alb < 3.0), n (%)	12 (15.4)	13 (23.6)	0.295
Diabetes, n (%)	17 (21.8)	12 (21.8)	0.997
Chronic lung disease, n (%)	20 (25.6)	10 (18.2)	0.311
MTX use, n (%)	33 (42.3)	16 (29.1)	0.120
GC use, n (%)	17 (21.8)	12 (21.8)	0.997
Follow up periods (month)	26.1±16.0	29.3 ± 15.6	0.240

RA: rheumatoid arthritis; RF: rheumatoid factor; ACPA: anti-citrullinated peptide antibody; CRP: c-reactive protein; DAS28: disease activity score 28; CDAI: clinical disease activity index; eGFR: estimated glomerular filtration rate; MTX: methotrexate; GC: glucocorticoid; * *p* < 0.05.

## Data Availability

The data presented in this study are available on request from the corresponding author. The data are not publicly available due to the information that could compromise the privacy of research participants.

## Abbreviations

JAKis	Janus kinase inhibitors
RA	Rheumatoid arthritis
AEs	adverse events
ACPA	anti-citrullinated protein/peptide antibody
bDMARD	Biological disease modifying anti rheumatic drug
MACE	major adverse cardiovascular events
MTX	methotrexate
RF	rheumatoid factor
CRP:	C-reactive protein
GC	glucocorticoid
